# Detection of circulating tumor cells with CK20 RT-PCR is an independent negative prognostic marker in colon cancer patients – a prospective study

**DOI:** 10.1186/s12885-016-3035-1

**Published:** 2017-01-13

**Authors:** Sebastian Hinz, Alexander Hendricks, Amke Wittig, Clemens Schafmayer, Jürgen Tepel, Holger Kalthoff, Thomas Becker, Christian Röder

**Affiliations:** 1 Department of General and Thoracic Surgery, University Hospital Schleswig-Holstein, Campus Kiel, Arnold-Heller Str. 7, 24105 Kiel, Germany; 2 Division Molecular Oncology, Institute for Experimental Cancer Research, Cancer Center North, University Hospital Schleswig-Holstein, Campus Kiel, Arnold-Heller Str. 7, 24105 Kiel, Germany; 3 Klinikum Osnabrück, Am Finkenhügel 1-3, 49076 Osnabrück, Germany

**Keywords:** Circulating tumor cells, CTC, DTC, CK20 RT-PCR, CK20, Colon carcinoma, EpCAM

## Abstract

**Background:**

Detection of circulating (CTC) or disseminated tumor cells (DTC) has been associated with negative prognosis and outcome in patients with colorectal cancer, though testing for these cells is not yet part of clinical routine. There are several different methodological approaches to detect tumor cells but standardized detection assays are not implemented so far.

**Methods:**

In this prospective monocentric study 299 patients with colon cancer were included. CTC and DTC were detected using CK20 RT-PCR as well as immunocytochemistry staining with anti-pan-keratin and anti-EpCAM antibodies. The primary endpoints were: Evaluation of CTC and DTC at the time of surgery and correlation with main tumor characteristics and overall (OS) and disease free survival (DFS).

**Results:**

Patients with detectable CTC had a 5-year OS rate of 68% compared to a 5-year OS rate of 85% in patients without detectable CTC in the blood (*p* = 0.002). Detection of DTC in the bone marrow with CK20 RT-PCR was not associated with a worse OS or DFS. Detection of pan-cytokeratin positive DTC in the bone marrow correlated with a significantly reduced 5-year OS rate (*p* = 0.048), but detection of DTC in the bone marrow with the anti-EpCAM antibody did not significantly influence the 5-year OS rate (*p* = 0.958). By multivariate analyses only detection of CTC with CK20 RT-PCR in the blood was revealed to be an independent predictor of worse OS (HR1.94; 95% CI 1.0–3.7; *p* = 0.04) and DFS (HR 1.94; 95% CI 1.1–3.7; *p* = 0.044).

**Conclusions:**

Detection of CTC with CK20 RT-PCR is a highly specific and independent prognostic marker in colon cancer patients. Detection of DTC in the bone marrow with CK20 RT-PCR or immunohistochemistry with anti-EpCAM antibody is not associated with a negative prognostic influence.

**Electronic supplementary material:**

The online version of this article (doi:10.1186/s12885-016-3035-1) contains supplementary material, which is available to authorized users.

## Background

Even though many efforts had been made in the past with regarding prevention, early diagnosis and also optimizing therapeutic strategies adenocarcinoma of the colon still poses a considerable clinical problem. With mortality being nearly half as high as the relatively high incidence of 51.7, it significantly contributes to cancer-related mortality in industrialized countries [1].

Long-term survival after putative complete tumor resection is mainly threatened by distant metastases, derived from circulating tumor cells. Hereby, tumor cells that can be detected in the peripheral blood are termed circulating tumor cells (CTC), whereas tumor cells found in the bone marrow are termed disseminated tumor cells (DTC). In particular the mechanisms, how cancer cells acquire the ability to seed out metastases in distant organs still pose one of the principal query in the treatment of advanced cancer. According to the “revisited” hypothesis of “seed and soil”, it does not only depend on the cell itself, but also on local environmental factors, whether circulating tumor cells can develop and grow out into liver and lung metastases [2]. To improve survival, systemic treatment is recommended for patients with proven lymph node metastases. However, conventional pathological staging criteria do lead to an underestimation of the actual tumor stage in nearly 25% of the patients as has been shown by sentinel lymph node mapping [3]. The dissemination of sole tumor cells, which may stand for the starting point of tumor recurrence, cannot be detected by conventional staging methods so far. However, initial studies demonstrated that immuno-cytological and molecular-biological techniques are able to identify disseminated tumor cells in the bone marrow, blood, peritoneal cavity and lymph nodes of cancer patients [4, 5]. Using the Polymerase Chain Reaction (PCR), increased sensitivity and more objective results could be reached [6]. It has been demonstrated in several studies that molecular biomarkers or high-risk gene signatures help to identify patients who are candidates of a worse clinical course [7], but with the exception of patients with mutated *KRAS* in metastatic colorectal cancer, predictive factors are still lacking [8].

Our analytical system assessed the ectopic expression by nested RT-PCR in blood and bone marrow of cytokeratin (CK) 20-mRNA, coding for an intermediate filament protein of epithelial cells. CK20 is expressed in gastrointestinal epithelial cells among others, as well as in tumors derived from these cells. The mRNA and protein can be detected in 97% of colon tumors [9]. Previously, we demonstrated that our CK20 nested RT-PCR assay is highly sensitive and specific [10], and also shows tumor stage-related detection rates in clinical samples [11].

The majority of studies analyzing the role of CTC have been including colon and rectal cancer patients in the same cohort summed as colorectal cancer patients as a whole. We have previously shown that in rectal cancer patients CTC detection by CK20 expression is not a prognostic marker, but a marker for response to neoadjuvant chemoradiation [12]. This finding even more stresses the biological differences and distinct modes of metastasis of colon and rectal cancer, which is underestimated in most clinical trials. Hence, we included only patients with colon cancer in this prospective study.

The presence of disseminated tumor cells can serve as an indicator for systemic disease at the time of primary tumor resection. Initial studies based on the immuno-cytochemical detection of cytokeratin-positive cells in blood or peritoneal lavage confirmed for the prognostic relevance of such minimal residual disease in otherwise R0-resected patients [13]. Several studies in patients with colorectal carcinoma employing either immuno-cytochemical methods or CK20 RT-PCR supported such findings in multivariate analyses in small cohorts of 53 and 90 patients, respectively [14, 15]. The prognostic significance of minimal residual disease in a larger multicenter trial of clinically relevant size remains to be shown.

During the last years detection of DTC and CTC with anti-EpCAM based detection systems has gained broad popularity. The CellSearch System (Veridex, Raritan, USA) has been approved for the detection of CTC in metastatic colorectal cancer [16] by the Food and Drug Administration (FDA) in the USA. Though a clear disadvantage of anti-EpCAM based detection systems is: A change in the expression profile during metastatic spread of tumor cells, which has already been reported as epithelial-mesenchymal transition (EMT) [17], may result in lower detection rates of CTC.

We investigated bone marrow and peripheral blood of colon carcinoma patients by CK20-specific nested RT-PCR after isolation of the mononuclear cell (PBMC) fraction and preparation of total RNA. In addition, DTC in bone marrow blood were analyzed in a subset of patients using immunocytochemistry with anti-pan-cytokeratin or anti-EpCAM antibodies. All patients underwent complete (R0) tumor resection and were subjected to a detailed clinical follow up. The primary endpoints of this study were: Evaluation of CTC and DTC at the time of surgery and correlation with main tumor characteristics and overall (OS) and disease free survival (DFS) in a large cohort of colon cancer patients with a reasonable long follow-up.

## Methods

### Patients

A total of 299 patients with colon cancer that underwent surgery at the Department of General and Thoracic Surgery, University Hospital Kiel, were sequentially included during a 7 year study period in this investigation. The study was approved by the local ethics committee of the Christian-Albrechts University, Kiel (A110/99) and all patients gave written informed consent prior to inclusion in the study. Patients with rectal cancer were not included. A total of 227 bone marrow and 299 venous blood samples were collected directly before skin incision and transferred to the laboratory for extraction of the mononuclear cells within 2 h. In all patients with stage IV disease (only liver metastases) the patients underwent synchronous liver resection. Only patients who underwent complete tumor (R0)-resection were included. Patients that underwent surgery for recurrent disease or had other malignancies were excluded from this study. Classification of the pathological tumor-stage and grade was performed at the Department of Pathology, University Hospital Schleswig-Holstein, Campus Kiel, according to the TNM-classification. The patient’s overall survival was one of the main endpoint result of our study. This was determined as the number of months between the date of surgery and the date of death or the date of the last follow up. Clinical follow-up was performed in cooperation with general practitioners and with the Cancer Registry of the Federal State of Schleswig-Holstein (Bad Segeberg, Germany). All individual data were obtained from the clinical research data base of the oncological biobank BMB-CCC of the Comprehensive Cancer Center Kiel and data were verified by re-examination of original patient records and of the PCR and immunocytochemistry results. Only patients with complete clinical data were considered for further analysis.

Patients with UICC-stage-III colon carcinoma were recommended to receive adjuvant chemotherapy and the vast majority did so. Patients developing recurrent disease during follow-up received either surgical treatment or palliative chemotherapy.

### Control group

The control collective (total *n* = 76 individuals) consisted of 38 healthy volunteers from whom peripheral venous blood samples (*n* = 38) were obtained. The volunteers were randomly recruited and not age/sex matched. Furthermore, 32 bone marrow samples and 30 venous blood samples were collected from a second group of 38 patients (6 bone marrow donors, 8 leukemia patients, and 24 patients with non-malignant diseases (liver cysts, liver adenoma, sigmoid diverticulitis, FAP, pancreatitis, hernias, ulcera ventriculi, primary sclerosing cholangitis). Part of this collective was already utilized and described in a previous report [11]. Informed written consent for participation in the study was obtained from all individuals of the control cohort and investigation of the samples was covered by the same approval of the local ethics committee as above for cancer patients.

### Sample collection, isolation of RNA and RT-PCR

Prior to surgery, 10 ml bone marrow blood was aspirated from the spina iliaca anterior under general anesthesia subsequent to a small cutaneous incision. Venous blood (20 ml) was taken in parallel from a central venous line. Lithium heparin was used as anti-coagulant. Fractions of mononuclear cells from blood or bone marrow were isolated by centrifugation through a Ficoll-Hypaque density cushion (GE Healthcare, Freiburg, Germany) according to the manufacturer’s recommendation. After washing in PBS, cells were counted, pelleted again, and subsequently centrifuged onto microscopic slides (cytospins) or lysed for RNA preparation with RNAPure reagent (PQLab, Erlangen, Germany) and further processed according to the manufacturer’s protocol. Total RNA was isolated and checked for integrity using a Bioanalyzer 2100 instrument (Agilent Technologies, Böblingen, Germany). CDNA synthesis and nested CK20 RT-PCR analysis was exactly performed as previously described in detail [11]. Every sample was assessed in triplicate. If at least one positive PCR test out of three was obtained, the sample was rated as CK20-positive. All assessments of PCR results were performed blinded, without knowledge of the clinical data.

### Immunocytochemistry

Mononuclear cell fractions from bone marrow blood were centrifuged as cytospins (Cytospin Centrifuge, Hettich, Germany) using 5x10^5^ cells per spot and slide. Slides were air-dried and stored dry and tightly sealed at -20 °C until further use. Cells were stained after 5 minutes aceton fixation, either with the primary pan-cytokeratin antibody A45-B/B3 detecting CK8, CK18 and CK19 (AS Diagnostik, Germany) or anti-EpCAM antibody BER-EP4 (Dako, Hamburg) using the Dako REAL detection system (Dako, Hamburg, Germany). Cytospins were analysed with an ACIS (automated cellular imaging system; Chromavision medical systems, St. Juan Capistrano, CA, USA) followed by manual microscopy by an independent scientist. Only positive cells with distinct morphological signs of a tumor cell were counted as positive cells [18]. Detection of at least one positive tumor cell regarded this patient as a positive case.

### Statistical analysis

Univariate Kaplan-Meier survival analysis was performed to compute the cumulative overall survival (OS) and disease free survial (DFS) rate in dependence on the CK20-RT-PCR status in blood and/or bone marrow and the positivity in immunocytochemistry, respectively. The detection rate of CTC and DTC and correlation with clinicopathologic parameters were analyzed with the χ^2^ test after crosstab analysis. Differences in the survival curves of the subgroups were assessed by the log-rank test. The Cox proportional-hazards model was used for multivariate analysis. Independence of categorical variables was tested by Pearson’s χ^2^ test after crosstab analysis. All reported P-values are two-sided and differences were judged significant if P was 0.05 or less. Calculations and tests were performed with SPSS 23.0 (SPSS Inc., Chicago, IL).

## Results

### Clinical characteristics

Our study population consisted of 299 patients with colon cancer. 108 patients (36.3%) underwent a right-sided hemicolectomy and 36 patients (12%) underwent a left–sided hemicolectomy. In 18 patients (6%) we performed a transverse-colon resection and in 122 patients (40.8%) a sigmoid resection was necessary. Fifteen patients (5%) were treated with a subtotal colectomy. All patients underwent open surgery. The mean age at the time of surgery was 67.4 years (range 29–92 years). The clinical and histological parameters are summarized in Table 1.

**Table 1:** Patients’ clinical and pathological characteristics and univariate analysis of factors influencing the 5-year overall survival (OS) and disease free survival (DFS) rate

Characteristics	Category	n	%	5y-OS (%)	*P*	5y-DFS (%)	*P*
CK20 blood	positive	112	37.4	68	**0.002**	78	**0.021**
(*n* = 299)	negative	187	62.6	85		89	
CK20 bone marrow	positive	81	35.7	71	0.09	85	0.419
(*n* = 227)	negative	146	63.3	79		86	
pan-cytokeratin	positive	30	22.4	59	**0.048**	61	**0.041**
(*n* = 134)	negative	104	77.6	76		80	
EpCAM	positive	12	19.7	55	0.958	44	0.548
(*n* = 61)	negative	49	80.3	64		72	
Sex	male	168	56.2	79	0.70	84	0.563
	female	131	43.8	77		87	
Age [years]	<70	160	53.5	82	**0.045**	88	0.332
	> 70	139	46.5	74		83	
UICC stage	I	87	29.1	98	**<0.001**	99	**<0.001**
	II	94	31.4	89		90	
	III	80	26.8	71		77	
	IV	38	12.7	24		48	
pT	T1	42	14.0	98	**<0.001**	98	**0.005**
	T2	70	23.4	89		94	
	T3	159	53.2	73		80	
	T4	28	9.4	47		75	
pN	N0	190	63.6	91	**<0.001**	93	**<0.001**
	N1	65	21.7	64		76	
	N2	44	14.7	46		64	
Grading	G1	21	7.0	95	0.054	76	0.704
	G2	236	78.9	79		85	
	G3	42	14.1	65		91	
Operation	right hemicolectomy	108	36.3				
	left hemicolectomy	36	12.0				
	transverse colon resection	18	6.0				
	sigmoid colon resection	122	40.8				
	subtotal colectomy	15	5.0				

The data in bold are regarded statistically significant (*p* < 0.05)

### Correlation of clinicopathologic characteristics and survival

The median follow-up was 55 months (range 4–168 months) and the 5-year overall survival (OS) rate for all patients included in the study was 78%. As expected, we found a strong correlation between tumor stage and OS. Furthermore, high pT-category and positive lymph node status predicted a highly significant worse 5-year OS and DFS rate (*p* < 0.001) (Table 1).

### Association of CTC and DTC detection with CK20 RT-PCR and clinicopathologic characteristics

The overall detection rate for circulating tumor cells in the blood (CTC) as determined by CK20 RT-PCR was 37.4% (Table 1). Higher tumor stage and pT category correlated with a higher detection rate of CTC by CK20 RT-PCR (*p* = 0.017 and *p* = 0.019, respectively), whereas the status of lymph node metastasis (pN) did not correlate with the detection rate of CTC or DTC (Table 2). A large number of patients who were treated for synchronous liver metastases combined with colon resection (pM1) were significantly positive for CTC in the blood (*p* = 0.002) (Table 2). Interestingly, we did not find any correlation between detection of disseminated tumor cells (DTC) by CK20 RT-PCR in the bone marrow and any clinicopathologic parameters analyzed (Table 2, right columns) although the general detection rate of DTC (35.7%) was nearly similar to the detection rate in the blood.

**Table 2:** Number of patients with CK20 positive tumor cells and association with patients’ characteristics

Characteristics	category	CK20+ BL	Detection rate (%)	*P*	CK20+BM	Detection rate (%)	*P*
All		112	37.4	-	81	35.7	-
Sex	male	60	35.7	0.50	42	31.3	0.101
	female	52	39.7		39	41.9	
Age [years]	< 70	62	38.7	0.636	38	32.7	0.216
	> 70	50	35.9		43	39.8	
UICC stage	I	28	32.2	**0.017**	20	31.7	0.633
	II	32	34.0		28	37.3	
	III	29	36.2		20	33.3	
	IV	23	60.5		13	44.8	
pT	T1	9	21.4	**0.019**	7	25.9	0.633
	T2	29	41.4		21	38.8	
	T3	58	36.5		44	35.7	
	T4	16	57.1		9	39.1	
pN	N0	66	34.7	0.134	51	34.9	0.646
	N1	24	36.9		20	40.8	
	N2	22	50.0		10	31.2	
Grading	G1	7	35.0	0.280	5	33.3	0.252
	G2	84	35.6		60	33.5	
	G3	21	48.8		16	48.4	
Liver Metastases	M0	89	35.1	**0.002**	68	34.3	0.271
	M1	23	62.1		13	44.8	

*BL* blood, *BM* bone marrow

The data in bold are regarded statistically significant (*p* < 0.05)

### Correlation analysis of survival and CTC and DTC detection by CK20-RT-PCR

**Fig 1:**
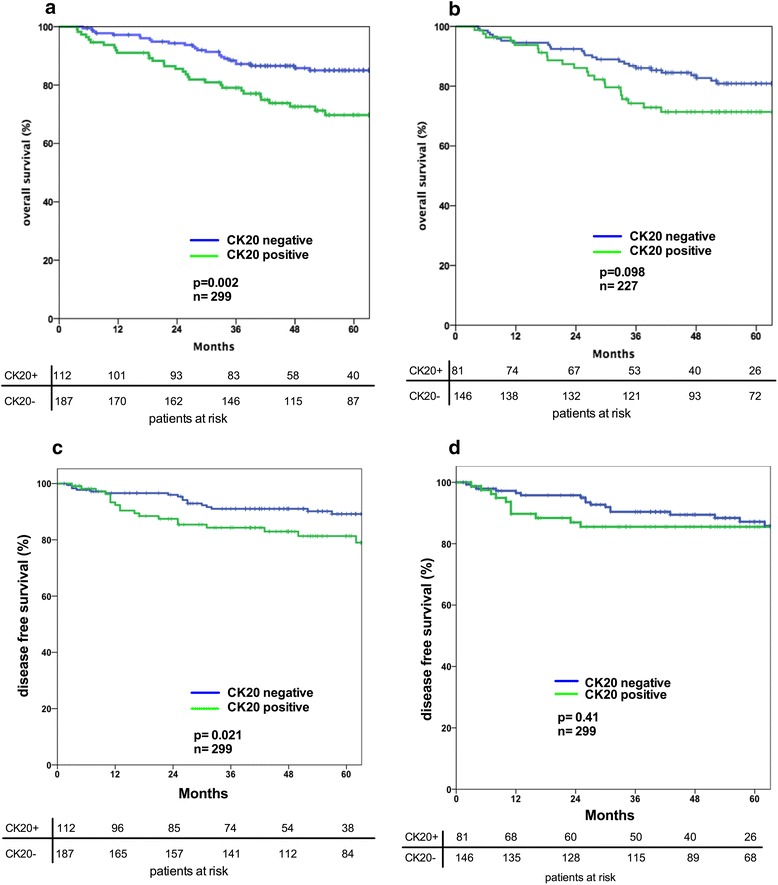
**a** Prognostic influence of the detection of circulating CK20+ tumor cells in the blood of colon cancer patients. **b** Prognostic influence of the detection of disseminated CK20+ tumor cells in the bone marrow of colon cancer patients. **c** Prognostic influence of the detection of circulating CK20+ tumor cells in the blood of colon cancer patients. **d** Prognostic influence of the detection of circulating CK20+ tumor cells in the bone marrow of colon cancer patients

Detection of CTC by CK20 RT-PCR in the blood of 299 patients was correlated with a significantly worse 5-year OS and DFS rate. Patients with detection of CTC had a 5-year OS rate of 68% compared to a 5-year OS rate of 85% in patients without detectable CTC in the blood (*p* = 0.002) (Fig. 1a, c). By contrast, analysis of bone marrow blood samples of 227 patients did not reveal a significant correlation between the CK20 expression status and the 5-year OS (*p* = 0.098) or DFS rate (*p* = 0.419) (Fig. 1b, d). During the follow-up period, 38 (12.7%) patients developed a recurrent disease. Patients with detectable CTC with CK20 RT-PCR had a significantly higher risk to develop a recurrent disease (20/38 patients, 52.6%) compared to the group without CTC (92/216, 35.2%) (*p* = 0.042, χ^2^ test after crosstab analysis). To further evaluate, if detection of CTC by CK20 RT-PCR is an applicable strategy to stratify CK20-positive high risk patients with UICC stage II disease against UICC Stage III patients without detectable CTC, we compared these two groups regarding detection rate of CK20 and the 5-year-OS or DFS rate. We did not find any significant differences with respect to detection rate or survival (data not shown).

### Control group

To determine the specificity of the CK20 RT-PCR we analyzed a control group of 76 individuals. This group consisted of blood samples from 38 healthy volunteers. In none of these healthy volunteers the CK20 RT-PCR was positive. Furthermore, we analyzed 32 bone marrow and 30 blood samples from a control group of 38 patients with different diseases (see Methods section). In these patients two bone marrow samples were tested positive for CK20. One patient had a familial adenomatosis polyposis (FAP) and underwent colectomy without detection of a colon cancer. The other patient suffered from a giant adenoma of the liver with a tumor mass of about 1.5 kg which was resected. This patient was also tested positive for CK20 in the blood. A second patient suffering from a chronic pancreatitis and undergoing pancreatic head resection was also tested positive for CK20 in the blood. Overall, none of the healthy controls were tested positive for CK20. The positive cases were already reported earlier by our group [11].

### Detection of DTC in the bone marrow by immunocytochemistry and correlation with clinicopathologic characteristics

As we could not observe any correlation of DTC detection in the bone marrow with CK20 RT-PCR and clinical parameters (Table 2) we additionally applied immunocytochemistry with two different antibodies to detect DTC in the bone marrow on the level of protein expression as an established alternative approach. The detection rate of DTC by immunocytochemistry was 22.3% with the pan-cytokeratin antibody A45-B/B 3 and 19.7% with anti-EpCAM antibody BER-EP4, respectively. The overall detection rate of DTC by immunocytochemistry was remarkably lower compared to CK20 RT-PCR. We could not demonstrate a correlation between the detection of DTC with pan-cytokeratin or anti-EpCAM antibody and any of the tested clinicopathologic parameters (Table 3).

**Table 3:** Number of patients with pan-cytokeratin or EpCAM positive tumor cells in the bone marrow detected with immunohistochemistry and association with patients’ characteristics (crosstabs, chi-square test, two sided)

Characteristics	Category	pan-cytokeratin	Detection rate (%)	*P*	EpCAM	Detection rate (%)	*P*
All		30 (134)	22.3	-	12 (61)	19.7	-
UICC stage	I	6 (30)	20.0	0.335	2 (8)	25.0	0.587
	II	7 (40)	17.5		2 (14)	14.2	
	III	8 (39)	20.5		3 (22)	13.6	
	IV	9 (25)	36.0		5 (17)	29.4	
pT	T1	4 (16)	25.0	0.861	2 (5)	40.0	0.575
	T2	4 (25)	16.0		1 (10)	10.0	
	T3	17 (5)	29.4		7 (34)	20.6	
	T4	5 (22)	22.7		2 (12)	16.4	
pN	N0	14 (75)	18.6	0.324	5 (24)	20.8	0.253
	N1	8 (35)	22.8		2 (21)	9.5	
	N2	8 (24)	33.3		5 (16)	31.2	
Grading	G1	1 (9)	11.1	0.702	0 (3)	0	0.108
	G2	23 (99)	23.23		12 (47)	25.5	
	G3	6 (26)	23.1		0 (11)	0	
Liver	M0	21 (104)	20.1	0.07	7 (44)	15.9	0.287
Metastases	M1	9 (30)	30		5 (17)	29.4	

### Correlation of survival and DTC detection by immunocytochemistry

**Fig. 2:**
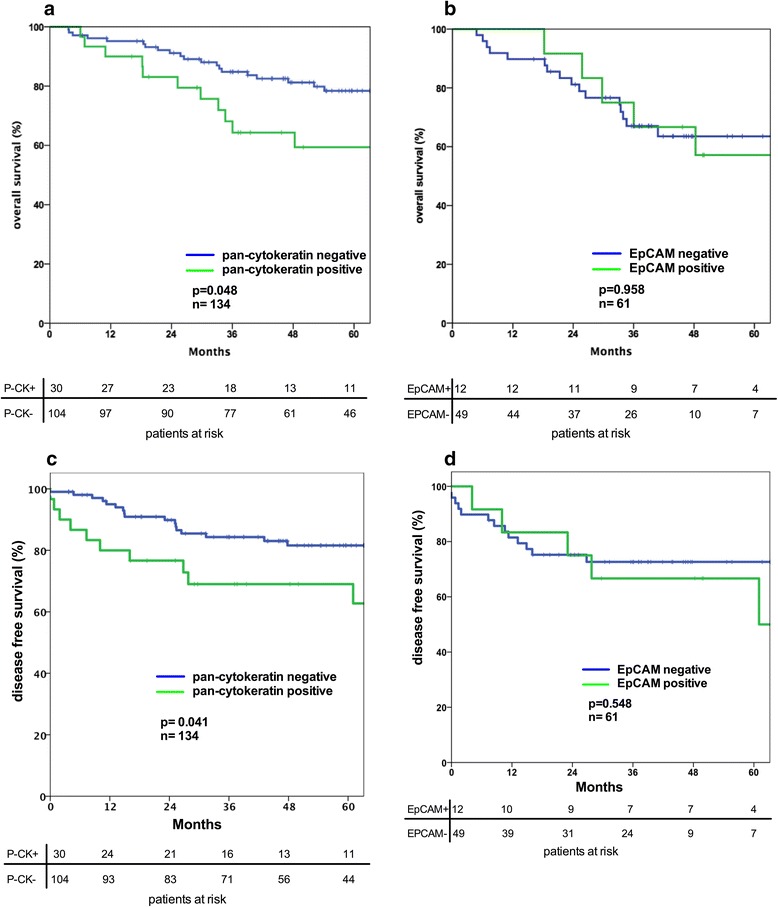
**a** Prognostic influence of the detection of pan-cytokeratin (A45-b/B3) positive tumor cells in the bone marrow of colon cancer patients. **b** Prognostic influence of the detection of EpCAM (BER-EP4) positive tumor cells in the bone marrow of colon cancer patients. **c** Prognostic influence of the detection of pan-cytokeratin (A45-b/B3) positive tumor cells in the bone marrow of colon cancer patients. **d** Prognostic influence of the detection of EpCAM (BER-EP4) positive tumor cells in the bone marrow of colon cancer patients

Detection of pan-cytokeratin positive DTC in the bone marrow was significantly correlated with a reduced 5-year OS rate of 59% compared to 76% in patients without cytokeratin positivity in the bone marrow (*p* = 0.048) (Fig. 2a). In line with this finding also the DFS was significantly reduced in patients with CK20-positive DTC in the bone marrow (*p* = 0.041) (Fig. 2c). Detection of DTC in the bone marrow with the anti-EpCAM antibody BER-EP4 did not significantly influence the 5-year OS (*p* = 0.958) or DFS rate (*p* = 0.548), respectively (Fig. 2b, d). Some exemplary immunohistochemistry stainings of pan-cytokeratin or anti-EpCAM positive DTC are shown in Additional file 1: Figure S1.

To further evaluate the relevance of DTC detection in the bone marrow, we combined detection of DTC in the bone marrow with either CK20 RT-PCR or immunocytochemistry (pan-cytokeratin or anti-EpCAM). With the combinational approach of these two different detection methods of DTC in the bone marrow we were able to increase the detection rate to 49.6% (62 of 125 patients positive with either technology/ antibody). The OS of the patients with detectable DTC in the bone marrow with either technology was not different from patients without DTC (*p* = 0.098).

### Multivariate cox regression analysis for independent factors influencing survival

All variables that showed a significant correlation in the univariate analysis were included in a Cox regression model. By multivariate analyses, detection of CTC by CK20-RT-PCR in the blood was revealed as an independent predictor of worse OS (HR1.94; 95% CI 1.0–3.7; *p* = 0.04). Higher UICC stage (HR 6.4; 95% CI 1.6–26.3; *p* = 0.01) and higher T stage (HR 3.3; 95% CI 1.3–8.4; *p* = 0.015) were also independent markers of worse OS. These markers were also independent predictors of an inferior DFS (Table 4).

**Table 4:** Multivariate Cox regression analysis of independent factors influencing overall and disease free survival

Factor	Category	Hazard ratio	95% CI	*P* value
Overall survival				
CK20 blood^a^	positive vs. negative	1.94	1.0–3.7	**0.042**
Age [years]	<70 vs. >70	2.7	1.4–5.4	**0.004**
pN stage	pN0 vs. pN1/2	0.7	0.2–2.7	0.62
pT stage	pT1/2 vs. pT3/4	3.3	1.3–8.4	**0.015**
UICC stage	I + II vs. III + IV	6.4	1.6–26.3	**0.01**
Disease free survival				
CK20 blood^a^	positive vs. negative	1.94	1.1–3.7	**0.044**
pN stage	pN0 vs. pN1/2	1.39	0.3–5.8	0.65
pT stage	pT1/2 vs. pT3/4	2.88	1.1–7.5	**0.03**
UICC stage	I + II vs. III + IV	4.9	1.0–23.7	**0.045**

*Abbreviation: CI* confidence interval

^a^Tumor cell detection with CK20 RT-PCR

The data in bold are regarded statistically significant (*p* < 0.05)

## Discussion

In this study we evaluated the role of CTC and DTC in colon cancer patients who were scheduled for potentially curative colon carcinoma resection. We show that CTC detection by CK20 RT-PCR is a highly sensitive and independent prognostic factor for OS and DFS in colon cancer patients.

In our study we applied two different technological approaches in parallel, i.e. RT-PCR and immunocytochemistry to detect CTC and DTC. Firstly, we employed a highly sensitive and specific nested CK20 RT-PCR to detect CTC and DTC. With this technique we were able to achieve detection rates of 37% in the blood and 35% in the BM. This technique is validly more sensitive than antibody-based detection of either intracellular protein markers (cytokeratins) or the cell surface EpCAM antigen, which yield detection rates of 22.3 and 19.7%, respectively for DTC in the bone marrow. For colorectal cancer patients in particular average detection rates of only 10.5% for CTC with the CellSearch™ system have been reported [19]. In addition to this, it has been demonstrated that the sensitivity of the qRT-PCR method is superior to immunomagnetic-based tools concerning detection of CTC in colorectal cancer patients [20].

Furthermore, we used immunocytochemistry to detect DTC with anti-pan-cytokeratin or anti-EpCAM antibodies. Using this methodological approach, we achieved detection rates of 22.3 and 19.7%, respectively. Recent reports have shown, that additionally incorporating CK20 RT-PCR as a biomarker, the sensitivity of the CellSearch^TM^ system could substantially be enhanced in colorectal cancer patients [21].

Though the major limitation of immunomagnetic enumeration platforms is, that only the subset of EpCAM+ CTC is detected. It has been shown, that a subgroup of CTC may exist, that has undergone epithelial to mesenchymal transition (EMT) and does not express EpCAM [22, 23]. Moreover, the cells that have encountered EMT have undergone dedifferentiation, increased cell mobility and have lost cell adhesions. These attributes make this subset of cells even more likely to have an aggressive metastatic potential and high drug resistance [24, 25].

In our study, we were able to show that disseminated tumor cells in the bone marrow have a different impact on overall survival than circulating tumor cells in the blood. Despite the combined detection rate for DTC in nearly 50% of the patients with either CK20 RT-PCR or immunocytochemistry the prognostic significance of DTC in the bone marrow was negligible compared to CTC in the peripheral blood. In clinical practice BM metastases are rarely seen in colon cancer. Solely in more advanced tumor stages, but what is the biological role of DTC in the bone marrow? This implies, that this organ might have a high ability to clear disseminated colon cancer cells or to prevent their proliferation. During the last years these findings have led to a hypothesis of tumor cell dormancy and tumor stem cells that reside in the bone marrow niche and recirculate after years to form distant metastases [26–28]. Recently, we have been able to show that patients with colorectal liver metastases and detectable DTC in the bone marrow at the time of liver surgery, had an unfavorable prognosis after complete liver metastases resection [29]. Interestingly, in this series of patients with apparent macro-metastases in the liver, CTC in the blood were not an additional negative prognostic marker. These findings support the hypothesis, that detection of DTC in the BM per se is not a negative prognostic factor, but only if under certain circumstances these dormant tumor cells re-circulate and consequently form solid organ metastases.

We included in our study exclusively patients with colon cancer as we have previously reported that in rectal cancer DTC and CTC have no prognostic influence on OS [12]. In accordance to our findings several other groups have also described that in rectal cancer CTC are not a prognostic factor for OS [30–32]. There are several clinical and biological hallmarks indicating that colon and rectal cancer are different with respect to anatomy, function and embryological origin [33, 34]. Furthermore, the treatment of primary non-metastasized colon and rectal cancer is different [35]. Future studies evaluating the role of circulating tumor cells should at least provide subgroup analysis of rectal and colon cancer patients.

The detection of CTC correlates with a higher T-category and the existence of liver metastases. In addition, patients with detectable CTC have a significantly higher risk to develop a recurrent disease. Interestingly, the detection of CTC did not correlate with lymph node metastases, which is in line with previous reports [19, 36]. Furthermore, in our study population we were not able to prove a prognostic influence of detectable CTC or DTC in early stage (UICC stage II) patients. As adjuvant therapy in patients without lymph node metastases remains a controversial issue, further molecular markers or risk factors are urgently needed to identify patients at risk for later metastases.

The biological significance of CTC or DTC is still uncertain. We and other groups can detect CTC in approx. 30% of T1-2 tumor patients [19, 37], but these patients have a very good prognosis. Recently, it has been shown with gene expression profiles of CTCs that there is a strong heterogeneity between the tumor cells. CTC are mostly dormant cells and disguised by the immune system, which may explain the low number of metastases opposing a high number of CTC in the blood flow [38]. It has been shown, that a subset of CTC express functional cancer stem cell characteristics [39]. Furthermore, in breast cancer a subset of metastases-initiating cells (MIC) among CTC was described that have a distinguished phenotype [40]. For the future, not the pure detection of DTC and CTC will be fundamental, but the quantification and phenotypic characterization of molecular markers of CTC that might allow selective targeting of the metastatic cascade of colon cancer.

## Conclusions

In our study we were able to show that detection of CTC with CK20 RT-PCR is a highly specific and independent prognostic marker in colon cancer patients. Patients with CTC in the blood had a significantly higher risk to develop a tumor recurrence during the follow-up. In contrast to this, detection of DTC in the bone marrow with CK20 RT-PCR or immunohistochemistry with anti-EpCAM antibody is not associated with a negative prognostic influence.

## Additional file

Additional file 1: Immunuohistochemistry staining of different bone marrow samples showing positive disseminated tumor cells from different tumor patients with colon cancer (scale bar 10μm). (TIFF 1522 kb)

## Abbreviations

BM: Bone marrow
CI: Confidence interval
CK: Cytokeratin
CTC: Circulating tumor cells
DFS: Disease free survival
DTD: Disseminated tumor cells
EMT: Epithelial-mesenchymal transition
FAP: Familial adenomatosis polyposis
FDA: Food and Drug Administration
HR: Hazard ratio
MIC: Metastases-initiating cell
OS: Overall survival
PCR: Polymerase chain reaction
