# Supplementary figures and images for: Detection of circulating tumor cells with CK20 RT-PCR is an independent negative prognostic marker in colon cancer patients – a prospective study
Source: BMC Cancer. 2017 Jan 13;17:53. doi: 10.1186/s12885-016-3035-1 (PMC5237158; doi:10.1186/s12885-016-3035-1)

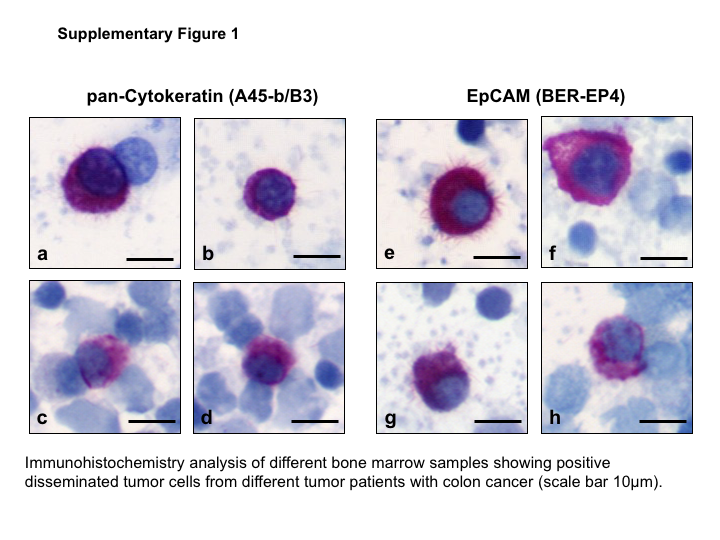

Supplement: Additional file 1: — Immunuohistochemistry staining of different bone marrow samples showing positive disseminated tumor cells from different tumor patients with colon cancer (scale bar 10μm). (TIFF 1522 kb) [file 12885_2016_3035_MOESM1_ESM.tiff]
